# *catena*-Poly[barium(II)-μ_2_-(dimethyl sulfoxide)-κ^2^ *O*:*O*-bis­(μ_2_-2,4,6-tri­nitro­phenolato-κ^4^ *O* ^2^,*O* ^1^:*O* ^1^,*O* ^6^)]

**DOI:** 10.1107/S2414314620014984

**Published:** 2020-11-13

**Authors:** Bikshandarkoil R. Srinivasan, Neha U. Parsekar, Kedar U. Narvekar

**Affiliations:** a School of Chemical Sciences, Goa University, Goa 403206, India; Vienna University of Technology, Austria

**Keywords:** crystal structure, barium, picrate anion, one-dimensional coordination polymer

## Abstract

The asymmetric unit of the title barium coordination polymer, [Ba(C_6_H_2_N_3_O_7_)_2_(C_2_H_6_OS)]_*n*_, consists of a barium cation (site symmetry *m*) and a dimethyl sulfoxide (DMSO) ligand (point group symmetry *m*) and a 2,4,6-tri­nitro­phenolate anion located in general positions. The S atom and the methyl group of DMSO are disordered over two sets of sites. The DMSO ligand bridges a pair of Ba^II^ atoms resulting in a chain extending parallel to the *a* axis. The unique 2,4,6-tri­nitro­phenolate anion also bridges a pair of Ba^II^ ions *via* the phenolic oxygen atom, with each Ba^II^ being additionally bonded to an oxygen atom of an adjacent nitro group. The *μ*_2_-monoatomic bridging binding mode of both types of ligands results in the formation of an infinite chain of face-sharing {BaO_10_} polyhedra flanked by the remaining parts of the 2,4,6-tri­nitro­phenolato and DMSO ligands. In the one-dimensional coordination polymer, parallel chains are inter­linked with the aid of C—H⋯O hydrogen bonds.

## Abstract 2

The Ba^II^ atom in the polymeric title compound, [Ba(C_2_H_6_OS)(C_6_H_2_N_3_O_7_)_2_], lies on a mirror plane and is coordinated by ten oxygen atoms, two of which stem from symmetry-related DMSO ligands and eight from four symmetry-related picrate anions.

## Graphical abstract

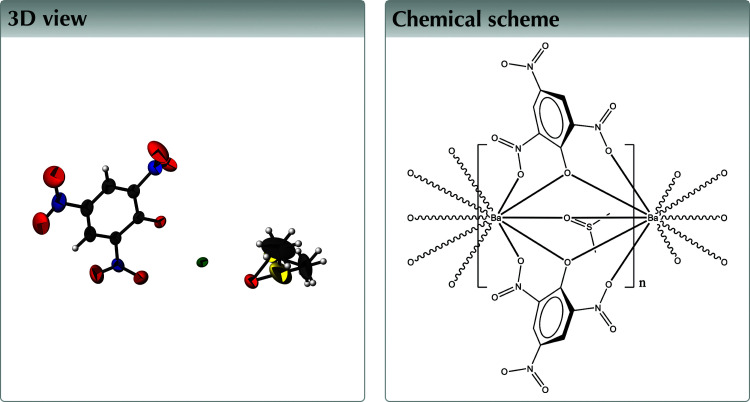

## Structure description

As part of an ongoing research program, we were investigating the synthetic and structural aspects of bivalent metal salts of picric acid (also known as 2,4,6-tri­nitro­phenol) containing zwitterionic glycine ligands (Srinivasan *et al.*, 2019 ▸). During the course of these studies, the glycine-free title compound, [Ba(C_6_H_2_N_3_O_7_)_2_(C_2_H_6_OS)] (**1**), was obtained serendipitously.

Compound (**1**) contains a coordinating DMSO mol­ecule but no glycine. A perusal of the Cambridge Structural Database (CSD, version 5.41, update November 2019; Groom *et al.*, 2016 ▸) reveals examples of structurally characterized Ba^II^ picrates devoid of DMSO (Hughes & Wingfield, 1977 ▸; Postma *et al.*, 1983 ▸; Chandler *et al.*, 1988 ▸; Harrowfield *et al.*, 1995 ▸; Hong *et al.*, 2007 ▸). In addition, Ba^II^ compounds containing DMSO as solvent mol­ecules (Studebaker *et al.*, 2000 ▸; Fichtel *et al.*, 2004 ▸; Ferrando-Soria *et al.*, 2012 ▸), and as monodentate and/or bridging bidentate ligands (Harrowfield *et al.*, 2004 ▸; Pi *et al.*, 2009 ▸; Gschwind & Jansen 2012 ▸) charge-balanced by anions other than picrate are also known. The title compound is a new example of a Ba^II^ compound in which both the DMSO and picrate ligands function as *μ*_2_-bridges.

**Figure 1:**
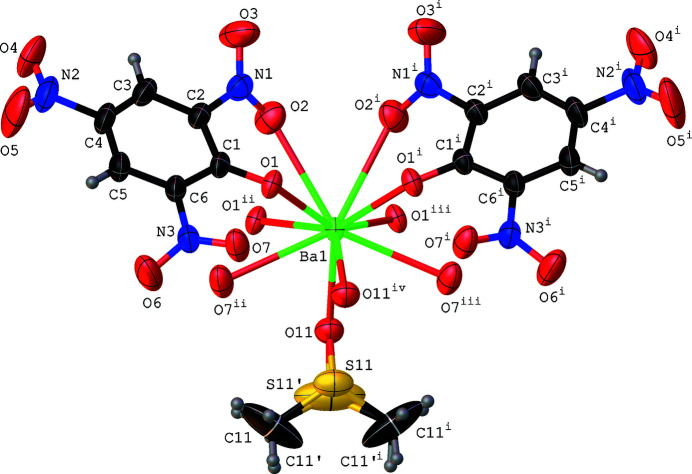
The coordination environment of the Ba^II^ atom in the crystal structure of [Ba(C_6_H_2_N_3_O_7_)_2_(C_2_H_6_OS)]. Displacement ellipsoids are drawn at the 50% probability level for non-hydrogen atoms. [Symmetry codes: (i) *x*, −*y* + 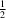 , *z*; (ii) *x* − 1, *y*, *z*; (iii) *x*, −*y* + 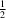 , *z*; (iv) *x* + 1, *y*, *z*.]

**Figure 2:**
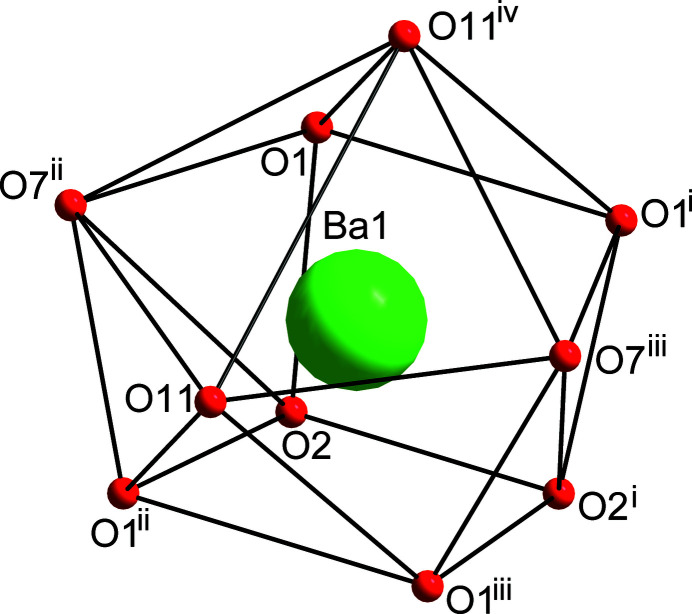
The distorted {BaO_10_} coordination polyhedron in the crystal structure of [Ba(C_6_H_2_N_3_O_7_)_2_(C_2_H_6_OS)]. Symmetry codes are as in Fig. 1 ▸.

**Figure 3:**
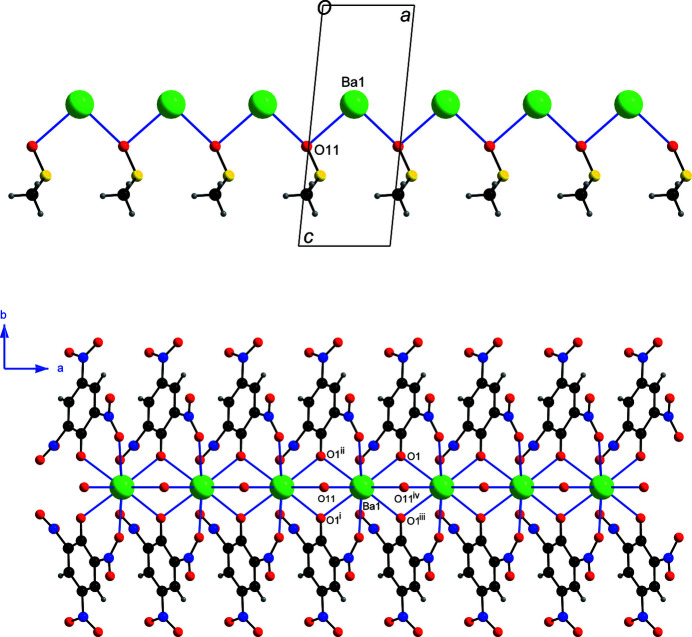
(Top) Ba^II^ cations bridged by O11 of DMSO, which results in the formation of chains extending along the *a*-axis direction. For clarity, the disordered S atom and the methyl group of the DMSO ligands as well as the picrate ligands are not displayed; (bottom) the chain showing the μ_2_-monoatomic bridging binding of the picrate and DMSO ligands. For clarity, only the bridging O11 atom of the DMSO ligands are shown. Each Ba^II^ atom in the chain is bonded to ten O atoms (see Fig. 2 ▸).

The asymmetric unit of (**1**) consists of a barium(II) cation and the S and O atom of a dimethyl sulfoxide (DMSO) ligand located on a mirror plane. The 2,4,6-tri­nitro­phenolate anion is located in a general position (Fig. 1 ▸). Atom S11 of the DMSO ligand and the attached methyl group (C11) are disordered over two sets of sites. Bond lengths and angles of the picrate anion and the DMSO ligand are in agreement with reported data (Srinivasan *et al.*, 2019 ▸, 2020 ▸). The central Ba^II^ atom exhibits ten-coordination and is bonded to eight oxygen atoms of four symmetry-related picrate anions and two oxygen atoms of two DMSO ligands resulting in a distorted {BaO_10_} polyhedron (Fig. 2 ▸). The deviation of the {BaO_10_} coordination polyhedron from a regular shape can be evidenced by the Ba—O bond lengths which range from 2.725 (2) to 2.970 (3) Å and the O—Ba—O bond angles which vary between 57.15 (12) and 151.94 (9)°. Both DMSO and picrate ligands exhibit an *μ*_2_-monoatomic bridging binding mode resulting in chains extending parallel to the *a* axis with an identical Ba⋯Ba separation of 4.1933 (2) Å (Fig. 3 ▸). The oxygen O11 atom of DMSO binds with a Ba^II^ atom at a Ba1—O11 distance of 2.906 (4) Å and further coordinates with a symmetry-related Ba^iv^ [symmetry code: (iv) *x* + 1, *y*, *z*] atom at a shorter distance of 2.783 (4) Å.

Binding of the nitro oxygen atom(s) of the picrate ligand is well documented in the literature for potassium picrate (Maartmann-Moe, 1969 ▸) and for many alkaline-earth picrates (Harrowfield *et al.*, 1995 ▸). In the mol­ecular compounds, [Ba(*L*)(pic)_2_] (*L* = dibenzo-24-crown-8), [Ba(acetone)(pic)_2_(phen)_2_] (pic = picrate; phen = 1,10-phenanthroline) and [Ba(*L*′)(pic)_2_] (*L*′ = di­aza 21-crown-7 ether), the picrate anion functions as a bidentate and or monodentate ligand (Hughes & Wingfield, 1977 ▸; Postma *et al.*, 1983 ▸; Chandler *et al.*, 1988 ▸). In the water-rich coordination polymer [Ba(H_2_O)_5_(C_6_H_2_N_3_O_7_)_2_]·H_2_O, one picrate anion functions as a bidentate ligand *via* the phenolate oxygen and an adjacent nitro O atom, while the second independent picrate anion functions as a *μ*_2_-bridg­ing tridentate ligand (Harrowfield *et al.*, 1995 ▸).

In the crystal structure of (**1**), the phenolate atom O1 makes a short Ba—O1 bond of 2.730 (2) Å and is further linked to a symmetry-related Ba^ii^ [symmetry code: (ii) *x* − 1, *y*, *z*] atom accompanied by the shortest Ba—O bond of 2.725 (2) Å. Each of the Ba^II^ atoms bridged by O1 is further coordinated by an oxygen atom of the nitro group with longer bond lengths [Ba1—O7^ii^ = 2.865 (2) Å; Ba1—O2 = 2.970 (3) Å]. Thus, the unique 2,4,6-tri­nitro­phenolate anion bridges a pair of Ba^II^ ions *via* the phenolic oxygen atom, and each Ba^II^ atom is bonded to an oxygen atom of an adjacent nitro group resulting in a *μ*_2_-monoatomic bridging bis-bidentate binding mode for this ligand. In the chain, each Ba^II^ atom is bonded to eight oxygen atoms of four symmetry-related picrate anions, and a pair of adjacent Ba^II^ atoms are bridged by two symmetry-related phenolate oxygen atoms (Fig. 3 ▸).

**Figure 4:**
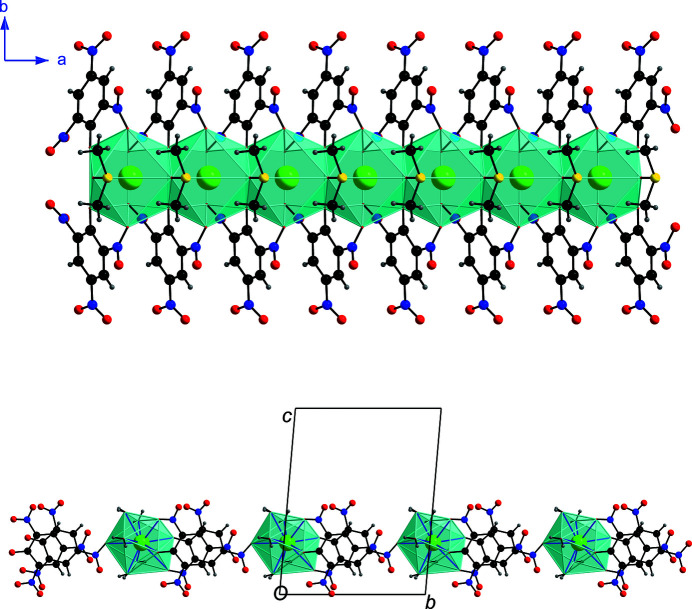
Face sharing {BaO_10_} polyhedra in the crystal structure of (**1**) (top) *versus* discrete {BaO_10_} polyhedra in the crystal structure of [Ba(H_2_O)_5_(C_6_H_2_N_3_O_7_)_2_]·H_2_O (bottom).

A polyhedral chain of face-sharing {BaO_9_} units flanked by organic ligands was reported recently in the one-dimensional polymeric compound [Ba(H_2_O)_2_(NMF)_2_(4-nba)_2_] (NMF = *N*-methyl­formamide; 4-nba = 4-nitro­benzoate) due to a *μ*_2_-binding aqua ligand and a pair of symmetry-related *μ*_2_-monoatomic bridging 4-nba ligands (Bhargao & Srinivasan, 2019 ▸). Likewise, the monoatomic bridging binding modes of the unique DMSO and the phenolate oxygen atoms of the picrate ligands in the structure of (**1**) result in the formation of an infinite chain of face-sharing {BaO_10_} polyhedra flanked by 2,4,6-tri­nitro­phenolate and dimethyl sulfoxide ligands (Fig. 4 ▸). In the reported water-rich compound [Ba(H_2_O)_5_(C_6_H_2_N_3_O_7_)_2_]·H_2_O, however, the central Ba^II^ atom exhibits ten-coordination and is bonded to five monodentate aqua ligands and a bidentate picrate anion (Harrowfield *et al.*, 1995 ▸). A second unique picrate anion is a *μ*_2_-bridging tridentate ligand and binds to a Ba^II^ atom *via* a phenolate oxygen atom. The cation is also linked to an oxygen atom of an *ortho* nitro group and is bridged to a second Ba^II^ *via* an oxygen of the nitro group *trans* to the phenolate oxygen (Fig. 4 ▸). In this one-dimensional coordination polymer, discrete {BaO_10_} polyhedra are bridged by a picrate anion due to the absence of any monoatomic bridge.

**Figure 5:**
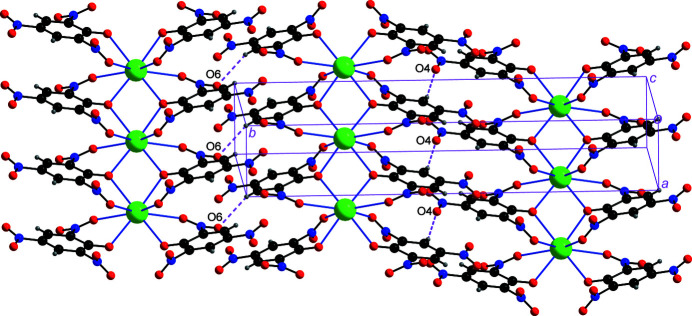
Inter­chain C—H⋯O hydrogen bonds, shown as broken pink lines for the C3—H3⋯O4^v^ inter­action on the right and for the C5—H5⋯O6^vi^ inter­action on the left, link adjacent polymeric chains. [Symmetry codes: (v) 1 − *x*, 1 − *y*, −*z*; (vi) 3 − *x*, 1 − *y*, 1 − *z*.]

The aromatic hydrogen atoms H3 and H5 are attached to the C3 and C5 donor atoms while the nitro oxygen atoms O4 and O6 function as hydrogen acceptors, resulting in inter­chain C—H⋯O hydrogen bonding inter­actions. In this way, each chain is linked on either side to two other chains (Table 1 ▸, Fig. 5 ▸) into a three-dimensional network.

## Synthesis and crystallization

To a slurry of barium carbonate (0.395 g, 2 mmol) in water, picric acid (0.916 g, 4 mmol) in water (40 ml) was added and the reaction mixture was heated on a water bath. Brisk effervescence was observed resulting in dissolution of the insoluble carbonate. The reaction mixture was then filtered into a beaker containing glycine (4 mmol, 0.3002 g) in water. The filtrate was left aside for crystallization. A yellow precipitate was filtered off and subsequently dissolved in DMSO (10 ml); this solution was left undisturbed. The crystalline product, which separated after two days, was isolated by filtration, washed with di­chloro­methane and dried in air; yield 0.95 g. Compound (**1**) can also be obtained without addition of glycine in the reaction by dissolving barium carbonate in aqueous picric acid to obtain the dipicrate of barium *in situ*. Concentration of the reaction mixture to a small volume followed by addition of DMSO afforded (**1**) as above.

## Refinement

Crystal data, data collection and structure refinement details are summarized in Table 2 ▸.

The S11 atom of the DMSO ligand and the attached methyl group (C11—H11) are disordered over two positions in a 0.73:0.27 ratio.

## Supplementary Material

Crystal structure: contains datablock(s) I, global. DOI: 10.1107/S2414314620014984/wm4142sup1.cif

Structure factors: contains datablock(s) I. DOI: 10.1107/S2414314620014984/wm4142Isup3.hkl

CCDC reference: 2043610

Additional supporting information:  crystallographic information; 3D view; checkCIF report

## Figures and Tables

**Table 1:** Hydrogen-bond geometry (Å, °)

*D*—H⋯*A*	*D*—H	H⋯*A*	*D*⋯*A*	*D*—H⋯*A*
C3—H3⋯O4^v^	0.93	2.43	3.283 (5)	153
C5—H5⋯O6^vi^	0.90 (4)	2.63 (4)	3.492 (5)	159 (3)

Symmetry codes: (v) 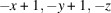 ; (vi) 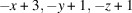 .

**Table 2:** Experimental details

Crystal data	
Chemical formula	[Ba(C_6_H_2_N_3_O_7_)_2_(C_2_H_6_OS)]
*M*_r_	671.68
Crystal system, space group	Monoclinic, *P*2_1_/*m*
Temperature (K)	293
*a*, *b*, *c* (Å)	4.1933 (2), 24.1526 (13), 11.0917 (7)
β (°)	95.775 (2)
*V* (Å^3^)	1117.66 (11)
*Z*	2
Radiation type	Mo *K*α
μ (mm^−1^)	1.96
Crystal size (mm)	0.23 × 0.16 × 0.05

Data collection	
Diffractometer	Bruker D8 Quest Eco
Absorption correction	Multi-scan (*SADABS*; Krause *et al.*, 2015 ▸)
*T*_min_, *T*_max_	0.537, 0.746
No. of measured, independent and observed [*I* > 2σ(*I*)] reflections	15884, 2860, 2696
*R*_int_	0.045
(sin θ/λ)_max_ (Å^−1^)	0.667

Refinement	
*R*[*F* ^2^ > 2σ(*F* ^2^)], *wR*(*F* ^2^), *S*	0.033, 0.087, 1.09
No. of reflections	2860
No. of parameters	182
H-atom treatment	H atoms treated by a mixture of independent and constrained refinement
Δρ_max_, Δρ_min_ (e Å^−3^)	1.73, −1.10

Computer programs: *APEX3* and *SAINT* (Bruker, 2019 ▸), *SHELXT* (Sheldrick, 2015*a* ▸), *SHELXL* (Sheldrick, 2015*b* ▸), *OLEX2* (Dolomanov *et al.*, 2009 ▸), *DIAMOND* (Brandenburg, 1999 ▸), *shelXle* (Hübschle *et al.*, 2011 ▸) and *publCIF* (Westrip, 2010 ▸).

## full crystallographic data

### Crystal data

**Table:**

[Ba(C_6_H_2_N_3_O_7_)_2_(C_2_H_6_OS)]	*F*(000) = 656
*M**_r_* = 671.68	*D*_x_ = 1.996 Mg m^−^^3^
Monoclinic, *P*2_1_/*m*	Mo *K*α radiation, λ = 0.71073 Å
*a* = 4.1933 (2) Å	Cell parameters from 9959 reflections
*b* = 24.1526 (13) Å	θ = 3.4–28.3°
*c* = 11.0917 (7) Å	µ = 1.96 mm^−^^1^
β = 95.775 (2)°	*T* = 293 K
*V* = 1117.66 (11) Å^3^	Plate, yellow
*Z* = 2	0.23 × 0.16 × 0.05 mm

### Data collection

**Table:**

Bruker D8 Quest Eco diffractometer	2696 reflections with *I* > 2σ(*I*)
Radiation source: Sealed Tube	*R*_int_ = 0.045
φ and ω scans	θ_max_ = 28.3°, θ_min_ = 3.1°
Absorption correction: multi-scan (*SADABS*; Krause *et al.*, 2015)	*h* = −5→5
*T*_min_ = 0.537, *T*_max_ = 0.746	*k* = −32→32
15884 measured reflections	*l* = −14→14
2860 independent reflections	

### Refinement

**Table:**

Refinement on *F*^2^	0 restraints
Least-squares matrix: full	Hydrogen site location: mixed
*R*[*F*^2^ > 2σ(*F*^2^)] = 0.033	H atoms treated by a mixture of independent and constrained refinement
*wR*(*F*^2^) = 0.087	*w* = 1/[σ^2^(*F*_o_^2^) + (0.055*P*)^2^ + 0.7054*P*] where *P* = (*F*_o_^2^ + 2*F*_c_^2^)/3
*S* = 1.09	(Δ/σ)_max_ < 0.001
2860 reflections	Δρ_max_ = 1.73 e Å^−^^3^
182 parameters	Δρ_min_ = −1.10 e Å^−^^3^

### Special details

**Table:**

Geometry. All esds (except the esd in the dihedral angle between two l.s. planes) are estimated using the full covariance matrix. The cell esds are taken into account individually in the estimation of esds in distances, angles and torsion angles; correlations between esds in cell parameters are only used when they are defined by crystal symmetry. An approximate (isotropic) treatment of cell esds is used for estimating esds involving l.s. planes.

### Fractional atomic coordinates and isotropic or equivalent isotropic displacement parameters (Å^2^)

**Table:**

	*x*	*y*	*z*	*U*_iso_*/*U*_eq_	Occ. (<1)
Ba1	0.45109 (5)	0.250000	0.41165 (2)	0.02648 (9)	
O1	0.9359 (5)	0.31686 (8)	0.3517 (2)	0.0342 (4)	
O2	0.4249 (7)	0.30881 (12)	0.1757 (3)	0.0558 (7)	
O3	0.6121 (10)	0.33930 (17)	0.0155 (3)	0.0842 (12)	
O4	0.7913 (9)	0.54083 (14)	0.0692 (4)	0.0824 (11)	
O5	1.1509 (12)	0.56275 (15)	0.2103 (4)	0.1123 (17)	
O6	1.3008 (10)	0.44197 (13)	0.5598 (3)	0.0835 (12)	
O7	1.4143 (7)	0.35863 (10)	0.5130 (2)	0.0522 (6)	
O11	−0.0224 (9)	0.250000	0.5858 (3)	0.0470 (8)	
N1	0.5951 (7)	0.33983 (13)	0.1240 (3)	0.0433 (6)	
N2	0.9650 (10)	0.52998 (14)	0.1609 (4)	0.0628 (9)	
N3	1.2850 (7)	0.40289 (11)	0.4887 (3)	0.0435 (6)	
C1	0.9409 (7)	0.36577 (11)	0.3105 (3)	0.0312 (5)	
C2	0.7786 (7)	0.38185 (13)	0.1958 (3)	0.0356 (6)	
C3	0.7865 (9)	0.43382 (14)	0.1459 (3)	0.0435 (7)	
H3	0.682230	0.441196	0.069627	0.052*	
C4	0.9522 (9)	0.47457 (14)	0.2118 (4)	0.0465 (8)	
C5	1.1114 (9)	0.46442 (14)	0.3244 (3)	0.0438 (7)	
H5	1.219 (10)	0.4924 (19)	0.364 (4)	0.053*	
C6	1.1081 (8)	0.41125 (12)	0.3708 (3)	0.0363 (6)	
S11	0.1576 (5)	0.250000	0.70846 (17)	0.0577 (7)	0.729 (6)
C11	0.031 (2)	0.3077 (4)	0.7858 (7)	0.156 (4)	0.73
H11A	0.143280	0.308937	0.865642	0.187*	0.73
H11B	0.076199	0.340762	0.742520	0.187*	0.73
H11C	−0.195036	0.305151	0.791823	0.187*	0.73
S11'	−0.079 (3)	0.250000	0.7161 (8)	0.143 (6)	0.271 (6)
C11'	0.031 (2)	0.3077 (4)	0.7858 (7)	0.156 (4)	0.27
H11D	−0.010490	0.305316	0.869186	0.187*	0.27
H11E	0.256229	0.313593	0.781421	0.187*	0.27
H11F	−0.087455	0.338061	0.747709	0.187*	0.27

### Atomic displacement parameters (Å^2^)

**Table:**

	*U*^11^	*U*^22^	*U*^33^	*U*^12^	*U*^13^	*U*^23^
Ba1	0.02267 (13)	0.02134 (13)	0.03503 (14)	0.000	0.00095 (8)	0.000
O1	0.0308 (10)	0.0254 (9)	0.0463 (12)	0.0007 (8)	0.0035 (8)	0.0095 (8)
O2	0.0552 (16)	0.0523 (16)	0.0588 (15)	−0.0154 (12)	0.0002 (12)	0.0058 (12)
O3	0.125 (3)	0.088 (3)	0.0385 (14)	−0.033 (2)	0.0037 (17)	0.0009 (15)
O4	0.102 (3)	0.0507 (18)	0.092 (2)	0.0103 (17)	−0.003 (2)	0.0391 (17)
O5	0.153 (4)	0.0435 (19)	0.131 (4)	−0.034 (2)	−0.032 (3)	0.034 (2)
O6	0.138 (3)	0.0460 (17)	0.0609 (18)	0.0187 (19)	−0.0173 (19)	−0.0189 (14)
O7	0.0638 (16)	0.0344 (12)	0.0545 (14)	0.0111 (11)	−0.0133 (12)	−0.0060 (10)
O11	0.056 (2)	0.048 (2)	0.0360 (16)	0.000	−0.0012 (14)	0.000
N1	0.0485 (16)	0.0407 (15)	0.0395 (13)	0.0030 (12)	−0.0011 (11)	0.0068 (12)
N2	0.081 (3)	0.0332 (16)	0.076 (2)	0.0000 (16)	0.013 (2)	0.0194 (16)
N3	0.0575 (17)	0.0280 (13)	0.0444 (14)	0.0025 (12)	0.0019 (12)	−0.0029 (11)
C1	0.0303 (13)	0.0235 (12)	0.0408 (14)	0.0041 (10)	0.0086 (11)	0.0049 (10)
C2	0.0379 (15)	0.0301 (14)	0.0393 (14)	0.0025 (11)	0.0062 (11)	0.0070 (11)
C3	0.0506 (19)	0.0359 (16)	0.0447 (16)	0.0058 (14)	0.0078 (14)	0.0135 (13)
C4	0.056 (2)	0.0278 (15)	0.057 (2)	0.0052 (14)	0.0117 (16)	0.0147 (14)
C5	0.055 (2)	0.0252 (14)	0.0520 (18)	−0.0005 (13)	0.0081 (15)	0.0026 (13)
C6	0.0420 (16)	0.0259 (13)	0.0415 (15)	0.0033 (11)	0.0070 (12)	0.0023 (11)
S11	0.0417 (12)	0.0901 (16)	0.0403 (9)	0.000	−0.0005 (7)	0.000
C11	0.141 (7)	0.213 (10)	0.110 (5)	0.007 (7)	−0.005 (5)	−0.117 (7)
S11'	0.092 (9)	0.285 (19)	0.054 (4)	0.000	0.011 (4)	0.000
C11'	0.141 (7)	0.213 (10)	0.110 (5)	0.007 (7)	−0.005 (5)	−0.117 (7)

### Geometric parameters (Å, º)

**Table:**

Ba1—O1^i^	2.725 (2)	N1—C2	1.461 (4)
Ba1—O1^ii^	2.725 (2)	N2—C4	1.456 (4)
Ba1—O1	2.730 (2)	N3—C6	1.451 (4)
Ba1—O1^iii^	2.730 (2)	C1—C6	1.432 (4)
Ba1—O11^iv^	2.783 (4)	C1—C2	1.435 (4)
Ba1—O7^ii^	2.865 (2)	C2—C3	1.373 (4)
Ba1—O7^i^	2.865 (2)	C3—C4	1.372 (5)
Ba1—O11	2.906 (4)	C3—H3	0.9300
Ba1—O2^iii^	2.970 (3)	C4—C5	1.379 (5)
Ba1—O2	2.970 (3)	C5—C6	1.384 (4)
Ba1—S11	3.629 (2)	C5—H5	0.90 (4)
Ba1—Ba1^iv^	4.1933 (2)	S11—C11^iii^	1.747 (7)
O1—C1	1.268 (3)	S11—C11	1.747 (7)
O2—N1	1.218 (4)	C11—H11A	0.9600
O3—N1	1.212 (4)	C11—H11B	0.9600
O4—N2	1.218 (5)	C11—H11C	0.9600
O5—N2	1.204 (6)	S11'—C11'	1.637 (9)
O6—N3	1.228 (4)	C11'—H11D	0.9600
O7—N3	1.217 (4)	C11'—H11E	0.9600
O11—S11	1.488 (4)	C11'—H11F	0.9600
O11—S11'	1.488 (9)		
			
O1^i^—Ba1—O1^ii^	72.68 (9)	O7^i^—Ba1—Ba1^iv^	95.35 (6)
O1^i^—Ba1—O1	151.94 (9)	O11—Ba1—Ba1^iv^	138.60 (7)
O1^ii^—Ba1—O1	100.46 (6)	O2^iii^—Ba1—Ba1^iv^	87.04 (6)
O1^i^—Ba1—O1^iii^	100.46 (6)	O2—Ba1—Ba1^iv^	87.04 (6)
O1^ii^—Ba1—O1^iii^	151.94 (9)	S11—Ba1—Ba1^iv^	115.50 (3)
O1—Ba1—O1^iii^	72.52 (9)	C1—O1—Ba1^iv^	126.78 (18)
O1^i^—Ba1—O11^iv^	136.32 (6)	C1—O1—Ba1	132.75 (18)
O1^ii^—Ba1—O11^iv^	136.32 (6)	Ba1^iv^—O1—Ba1	100.46 (6)
O1—Ba1—O11^iv^	67.10 (7)	N1—O2—Ba1	137.6 (2)
O1^iii^—Ba1—O11^iv^	67.10 (7)	N3—O7—Ba1^iv^	139.3 (2)
O1^i^—Ba1—O7^ii^	124.46 (7)	S11—O11—Ba1^ii^	158.2 (2)
O1^ii^—Ba1—O7^ii^	58.69 (7)	S11—O11—Ba1	106.9 (2)
O1—Ba1—O7^ii^	67.95 (8)	S11'—O11—Ba1	146.2 (5)
O1^iii^—Ba1—O7^ii^	135.08 (7)	Ba1^ii^—O11—Ba1	94.94 (9)
O11^iv^—Ba1—O7^ii^	78.34 (6)	O3—N1—O2	123.9 (3)
O1^i^—Ba1—O7^i^	58.69 (7)	O3—N1—C2	117.9 (3)
O1^ii^—Ba1—O7^i^	124.46 (7)	O2—N1—C2	118.2 (3)
O1—Ba1—O7^i^	135.08 (7)	O5—N2—O4	123.0 (4)
O1^iii^—Ba1—O7^i^	67.95 (8)	O5—N2—C4	118.4 (4)
O11^iv^—Ba1—O7^i^	78.34 (6)	O4—N2—C4	118.6 (4)
O7^ii^—Ba1—O7^i^	132.66 (11)	O7—N3—O6	122.6 (3)
O1^i^—Ba1—O11	65.44 (7)	O7—N3—C6	119.9 (3)
O1^ii^—Ba1—O11	65.44 (7)	O6—N3—C6	117.5 (3)
O1—Ba1—O11	137.49 (6)	O1—C1—C6	124.8 (3)
O1^iii^—Ba1—O11	137.48 (6)	O1—C1—C2	123.3 (3)
O11^iv^—Ba1—O11	94.94 (9)	C6—C1—C2	111.9 (3)
O7^ii^—Ba1—O11	70.84 (6)	C3—C2—C1	125.2 (3)
O7^i^—Ba1—O11	70.84 (6)	C3—C2—N1	116.6 (3)
O1^i^—Ba1—O2^iii^	62.84 (7)	C1—C2—N1	118.2 (3)
O1^ii^—Ba1—O2^iii^	96.33 (7)	C4—C3—C2	118.2 (3)
O1—Ba1—O2^iii^	91.77 (8)	C4—C3—H3	120.9
O1^iii^—Ba1—O2^iii^	57.66 (7)	C2—C3—H3	120.9
O11^iv^—Ba1—O2^iii^	124.61 (8)	C3—C4—C5	121.9 (3)
O7^ii^—Ba1—O2^iii^	141.74 (8)	C3—C4—N2	119.3 (3)
O7^i^—Ba1—O2^iii^	84.78 (8)	C5—C4—N2	118.8 (4)
O11—Ba1—O2^iii^	128.20 (8)	C4—C5—C6	118.7 (3)
O1^i^—Ba1—O2	96.33 (7)	C4—C5—H5	119 (3)
O1^ii^—Ba1—O2	62.84 (7)	C6—C5—H5	123 (3)
O1—Ba1—O2	57.66 (7)	C5—C6—C1	124.1 (3)
O1^iii^—Ba1—O2	91.77 (8)	C5—C6—N3	116.1 (3)
O11^iv^—Ba1—O2	124.61 (8)	C1—C6—N3	119.7 (3)
O7^ii^—Ba1—O2	84.78 (8)	O11—S11—C11^iii^	107.4 (3)
O7^i^—Ba1—O2	141.74 (8)	O11—S11—C11	107.4 (3)
O11—Ba1—O2	128.20 (8)	C11^iii^—S11—C11	105.9 (7)
O2^iii^—Ba1—O2	57.15 (12)	O11—S11—Ba1	50.03 (16)
O1^i^—Ba1—S11	83.59 (5)	C11^iii^—S11—Ba1	126.4 (4)
O1^ii^—Ba1—S11	83.59 (5)	C11—S11—Ba1	126.4 (4)
O1—Ba1—S11	123.35 (5)	S11—C11—H11A	109.5
O1^iii^—Ba1—S11	123.35 (5)	S11—C11—H11B	109.5
O11^iv^—Ba1—S11	71.83 (8)	H11A—C11—H11B	109.5
O7^ii^—Ba1—S11	66.89 (6)	S11—C11—H11C	109.5
O7^i^—Ba1—S11	66.89 (6)	H11A—C11—H11C	109.5
O11—Ba1—S11	23.10 (8)	H11B—C11—H11C	109.5
O2^iii^—Ba1—S11	144.44 (6)	O11—S11'—C11'	113.2 (5)
O2—Ba1—S11	144.44 (6)	S11'—C11'—H11D	109.5
O1^i^—Ba1—Ba1^iv^	140.18 (4)	S11'—C11'—H11E	109.5
O1^ii^—Ba1—Ba1^iv^	140.18 (4)	H11D—C11'—H11E	109.5
O1—Ba1—Ba1^iv^	39.73 (4)	S11'—C11'—H11F	109.5
O1^iii^—Ba1—Ba1^iv^	39.73 (4)	H11D—C11'—H11F	109.5
O11^iv^—Ba1—Ba1^iv^	43.66 (7)	H11E—C11'—H11F	109.5
O7^ii^—Ba1—Ba1^iv^	95.35 (6)		
			
Ba1—O2—N1—O3	144.7 (4)	O5—N2—C4—C5	10.7 (7)
Ba1—O2—N1—C2	−38.2 (5)	O4—N2—C4—C5	−170.5 (4)
Ba1^iv^—O7—N3—O6	173.9 (3)	C3—C4—C5—C6	1.3 (6)
Ba1^iv^—O7—N3—C6	−7.2 (6)	N2—C4—C5—C6	−177.8 (3)
Ba1^iv^—O1—C1—C6	−58.8 (4)	C4—C5—C6—C1	−1.9 (5)
Ba1—O1—C1—C6	119.2 (3)	C4—C5—C6—N3	178.8 (3)
Ba1^iv^—O1—C1—C2	120.7 (3)	O1—C1—C6—C5	179.9 (3)
Ba1—O1—C1—C2	−61.3 (4)	C2—C1—C6—C5	0.3 (4)
O1—C1—C2—C3	−177.7 (3)	O1—C1—C6—N3	−0.8 (5)
C6—C1—C2—C3	1.9 (4)	C2—C1—C6—N3	179.6 (3)
O1—C1—C2—N1	0.9 (4)	O7—N3—C6—C5	−147.6 (3)
C6—C1—C2—N1	−179.5 (3)	O6—N3—C6—C5	31.4 (5)
O3—N1—C2—C3	39.9 (5)	O7—N3—C6—C1	33.0 (5)
O2—N1—C2—C3	−137.3 (3)	O6—N3—C6—C1	−148.0 (4)
O3—N1—C2—C1	−138.8 (4)	Ba1^ii^—O11—S11—C11^iii^	56.7 (4)
O2—N1—C2—C1	44.0 (4)	Ba1—O11—S11—C11^iii^	−123.3 (4)
C1—C2—C3—C4	−2.5 (5)	Ba1^ii^—O11—S11—C11	−56.7 (4)
N1—C2—C3—C4	178.9 (3)	Ba1—O11—S11—C11	123.3 (4)
C2—C3—C4—C5	0.7 (6)	Ba1^ii^—O11—S11—Ba1	180.000 (2)
C2—C3—C4—N2	179.8 (3)	Ba1^ii^—O11—S11'—C11'	−112.1 (7)
O5—N2—C4—C3	−168.4 (5)	Ba1—O11—S11'—C11'	67.9 (7)
O4—N2—C4—C3	10.4 (6)		

Symmetry codes: (i) *x*−1, −*y*+1/2, *z*; (ii) *x*−1, *y*, *z*; (iii) *x*, −*y*+1/2, *z*; (iv) *x*+1, *y*, *z*.

### Hydrogen-bond geometry (Å, º)

**Table:**

*D*—H···*A*	*D*—H	H···*A*	*D*···*A*	*D*—H···*A*
C3—H3···O4^v^	0.93	2.43	3.283 (5)	153
C5—H5···O6^vi^	0.90 (4)	2.63 (4)	3.492 (5)	159 (3)

Symmetry codes: (v) −*x*+1, −*y*+1, −*z*; (vi) −*x*+3, −*y*+1, −*z*+1.
